# A Data-Driven Mathematical Model of CA-MRSA Transmission among Age Groups: Evaluating the Effect of Control Interventions

**DOI:** 10.1371/journal.pcbi.1003328

**Published:** 2013-11-21

**Authors:** Xiaoxia Wang, Sarada Panchanathan, Gerardo Chowell

**Affiliations:** 1 Mathematical, Computational and Modeling Sciences Center, School of Human Evolution and Social Change, Arizona State University, Tempe, Arizona, United States of America; 2 Department of Pediatrics, Maricopa Integrated Health System, Phoenix, Arizona, United States of America; 3 Division of International Epidemiology and Population Studies, Fogarty International Center, National Institutes of Health, Bethesda, Maryland, United States of America; 4 Department of Biomedical Informatics, Arizona State University, Tempe, Arizona, United States of America; Pennsylvania State University, United States of America

## Abstract

Community associated methicillin-resistant *Staphylococcus aureus* (CA-MRSA) has become a major cause of skin and soft tissue infections (SSTIs) in the US. We developed an age-structured compartmental model to study the spread of CA-MRSA at the population level and assess the effect of control intervention strategies. We used Monte-Carlo Markov Chain (MCMC) techniques to parameterize our model using monthly time series data on SSTIs incidence in children (≤19 years) during January 2004 -December 2006 in Maricopa County, Arizona. Our model-based forecast for the period January 2007–December 2008 also provided a good fit to data. We also carried out an uncertainty and sensitivity analysis on the control reproduction number, 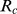 which we estimated at 1.3 (95% CI [1.2,1.4]) based on the model fit to data. Using our calibrated model, we evaluated the effect of typical intervention strategies namely reducing the contact rate of infected individuals owing to awareness of infection and decolonization strategies targeting symptomatic infected individuals on both 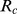 and the long-term disease dynamics. We also evaluated the impact of hypothetical decolonization strategies targeting asymptomatic colonized individuals. We found that strategies focused on infected individuals were not capable of achieving disease control when implemented alone or in combination. In contrast, our results suggest that decolonization strategies targeting the pediatric population colonized with CA-MRSA have the potential of achieving disease elimination.

## Author Summary

Community associated methicillin-resistant *Staphylococcus aureus* (CA-MRSA) is a bacteria that causes skin infections in the US. We developed a mathematical model of CA-MRSA transmission among different age groups at the population level. We parameterized the model using monthly time series data on number of SSTIs in children during the period January 2004–December 2006 in Maricopa County, Arizona. Our model-based forecast to additional time series data covering the period 2007–2008 yielded a good fit to data. Using our calibrated model, we calculated that an infected individual generates on average 1.3 infected people in a totally susceptible population in the study area. We assessed the impact of intervention strategies including reductions in contact rates between infected and non-infected individuals and the effect of decolonization strategies aimed at infected individuals by drug treatment, and found that neither of the two strategies when implemented alone or in combination were able to control the disease. In contrast, we found that decolonization strategies targeting the pediatric population colonized with CA-MRSA have the potential of achieving disease elimination.

## Introduction

### Background

*Staphylococcus aureus* is one of the most common bacterial pathogens in humans and the most frequent cause of skin and soft tissue infections (SSTIs) [1]. Strains of health care-associated methicillin-resistant *S. aureus* (HA-MRSA) were first identified among hospitalized patients in 1960 [2] and dominated MRSA infections until late 1980s. Since the early 1990s, there has been a dramatic increase in community-associated MRSA (CA-MRSA), which is now endemic at unprecedented levels in many regions in the US [3]–[6]. This increase in CA-MRSA appears to have regional variation and is more pronounced in children compared to adults [4], [5], [7]. Cases of HA-MRSA and CA-MRSA are characterized by significantly different epidemiological and microbiological features [8].

Evidence indicates that CA-MRSA infections result from physical contacts with MRSA carriers at home, community facilities such as gyms, nursing homes, or kindergartens [9]. In addition to regular treatment of the actual infection of the infected individuals, typical intervention strategies against CA-MRSA related SSTIs focus on pharmaceutical treatment via decolonization using mupirocin [10]–[12] and reductions in contact rates between infected and non-infected individuals. However, the role of these control interventions on CA-MRSA transmission dynamics remains poorly understood. In particular, in this paper we asked if control strategies targeting symptomatic infected individuals were sufficient to achieve disease control in a population.

Although several mathematical models for MRSA transmission in hospital settings, nursing homes, and other inpatient facilities have been developed (e.g., [13]–[18]), there is a scarcity of transmission models of MRSA and relevant epidemiological data to parameterize them at the community level, but these could be useful to elucidate the transmission dynamics and the effect of control interventions on CA-MRSA. For instance, in [13], [14] and [19], compartmental transmission models were developed to study the invasion of CA-MRSA into hospitals and the likelihood of coexistence between CA-MRSA and hospital-acquired (HA-) MRSA. Moreover, HA-MRSA dynamics were exclusively studied in both [16] and [17]. In these studies, health care workers were modeled as vectors transmitting disease among patients (or residents) with the goal of assessing the impact of quantifying the effect of targeted control measures. Furthermore, most of these modeling studies have assumed homogenous mixing, but recent work has pointed to age-specific variation in MRSA infection risk [4], [20]. Hence, age-structured CA-MRSA transmission models at the community level and tailored to local epidemiological data could increase our understanding of the transmission dynamics of CA-MRSA and the impact of routine and novel intervention strategies.

Here we developed and parameterized an age-structured compartmental transmission model to study the transmission dynamics of CA-MRSA at the population level and evaluate the effect of various intervention strategies. To calibrate the model, we employed a unique dataset Dataset S1) covering several years of SSTIs incidence during the period January 2004–December 2006 in Maricopa County, Arizona. We also used additional incidence data for subsequent years 2007–2008 for validation purposes. Based on our calibrated model, we estimated the reproduction number denoting the average number of secondary infections generated by primary infectious individual [21]–[23] and evaluated the effect of contact rate reductions aimed at infected individuals owing to awareness of infection as well as decolonization treatment strategies targeting symptomatic infected individuals or the general (asymptomatic) colonized subpopulation.

### Epidemiological data

We obtained detailed data on CA-MRSA infections from the Center for Health Information Research (CHIR), which is a university-community partnership between Arizona State University and several Arizona providers, insurers and employers. The dataset (Dataset S1) comprises records on hospitalization and outpatients visits by children and teenagers (age≤19 years) enrolled in the Medicaid program of Arizona from January 1, 2004 to December 31, 2008. We extracted records of all encounters diagnosed with skin or soft tissue infection (SSTI) based on ICD 9 codes (680.xx-682.9x). Each record contains information about the type of infection (first-time infection or recurrent infection), age group, month and year of hospital or clinic visit, and whether the patient was treated with mupirocin. Our data are based on SSTIs related infections, with a stationary fraction of MRSA-related infections during our study period [24]. We also obtained population data by age groups for our study setting. An extended data description is given in Text S1.

## Methods

### Model description

**Figure 1:**
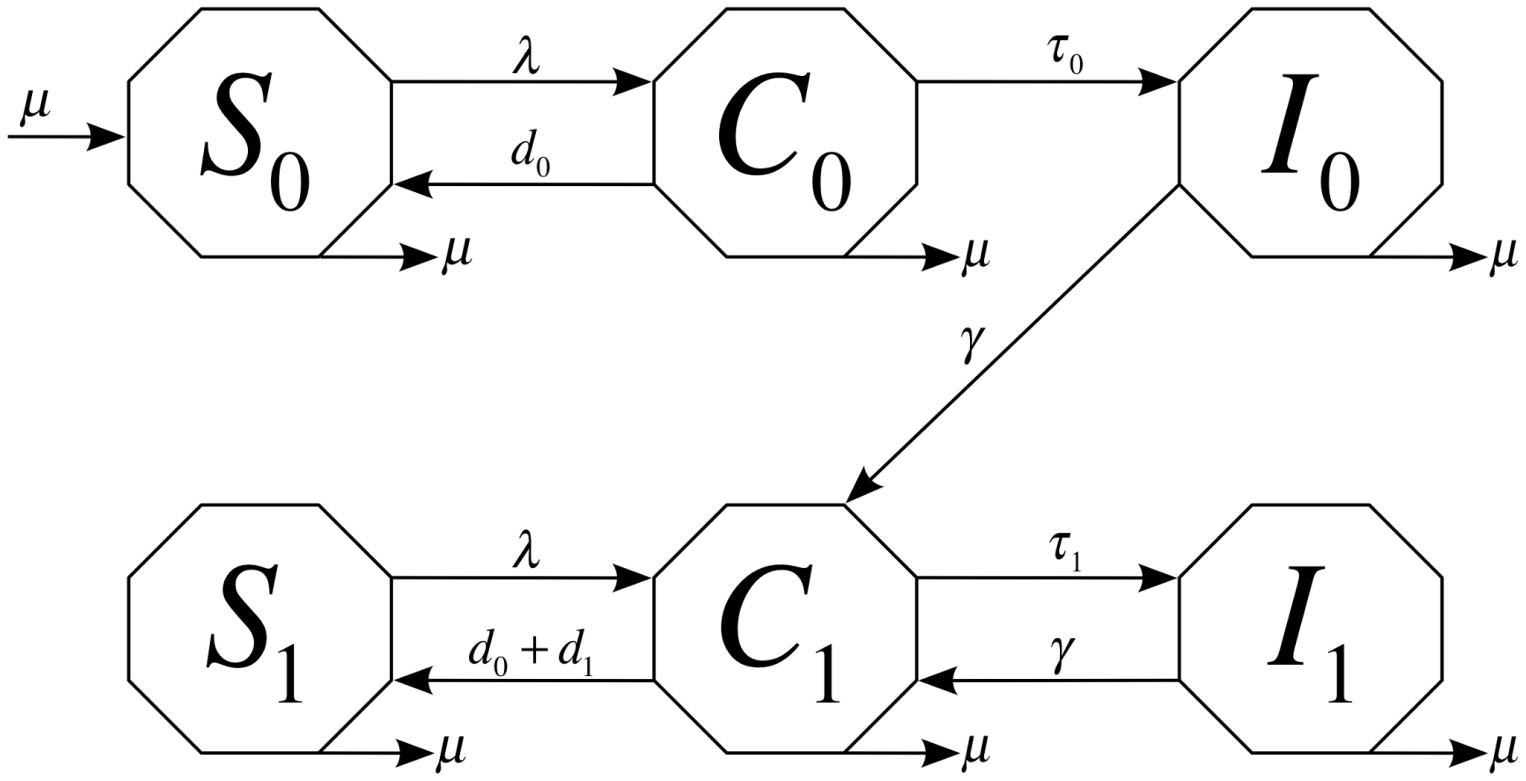
Flow diagram for the compartment model of the transmission dynamics of CA-MRSA for each age group. 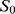
 is the number of susceptible people with no prior colonization with CA-MRSA, 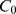 is the number of people colonized for the first time, and 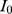 are the number of people with first-time infection. 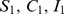 are individuals with a history of past infections. People with infections from both groups (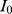 and 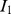) become colonized (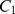) with a common cure rate (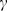). Further decolonization treatment (e.g. mupirocin) clears individuals and progress to the susceptible state (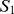). Susceptible individuals with or without past infections progress to the colonized stage with the same force of infection 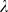, but their progression rate from colonized to infected stages are different (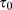 vs 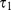, respectively). Colonized individuals without past infections have only spontaneous decolonization with rate 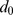 while those with past infections are subject to additional decolonization treatment with rate 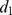. 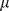 is the population immigration/migration rate per month.

We developed an SEIS (Susceptible-Exposed-Infected-Susceptible) transmission model that incorporates age heterogeneity in contact rates, infectiousness, and decolonization treatment rates. Our model also keeps track of individuals with past infections because these individuals have been observed to have a higher rate of infection compared to those with no past infections [24], and we are interested in assessing the effect of age-specific variation in infectiousness and the effect of targeted interventions. Let 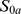, 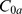, 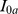 be the respective number of susceptible, colonized (asymptomatic), and infected (symptomatic) individuals in age group 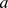 (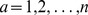) with no prior infections. Similarly, let 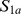, 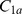, 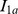 be the corresponding epidemiological states for individuals with prior infection history. The schematic view of transitions among the 6 epidemiological states in our model for each age group is shown in Figure 1. Mupirocin is used for decolonization of CA-MRSA patients who have been treated by other antibiotics. Hence, in our model only decolonized individuals coming from the infected compartments experience the additional decolonization rate 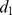. Our transmission model is given by the following system of differential equations:
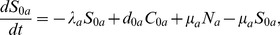
(1a)

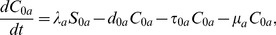
(1b)

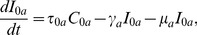
(1c)

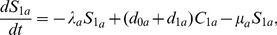
(1d)

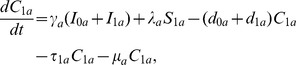
(1e)

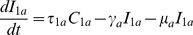
(1f)for *a* = 1, 2, …, *n*. The spontaneous (natural) decolonization rate per unit of time (month is denoted by *d*_0_) and is assumed to be the same for all colonized individuals independently of their infection history. The progression rate from colonized to infected is *τ*0 for those colonized for the first time and *τ*1 for those with prior infections, where *τ*1>*τ*0, with a relative risk factor 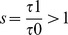. Infected individuals in compartments *I*_0_ and *I*_1_ progress to the colonization (with prior infections) stage (*C*1) following antibiotic treatment at a common cure rate *γ*. Further, treatment for decolonization aimed at *C*1 transfers individuals to compartment *S*1 at a decolonization rate *d*_1_. Both colonized and infected individuals contribute to the force of infection, which is given by
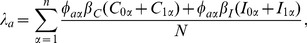
(1g)where 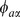 denotes the contact rate for the colonized and infected individuals with susceptible individuals in age group 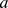. Moreover, 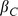 and 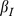 are the probabilities of transmission per contact (contagiousness) for colonized and infected individuals, respectively, which are assumed to be invariant across age groups. Because infected individuals are assumed to be more infectious than colonized individuals, 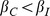, with a relative contagiousness factor 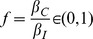. Further, 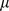 is the population immigration/migration rate and 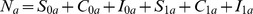 is the total population size with new recruits into the susceptible population with no past CA-MRSA infection history.

### Parameters

#### Age-specific contact rates

**Figure 2:**
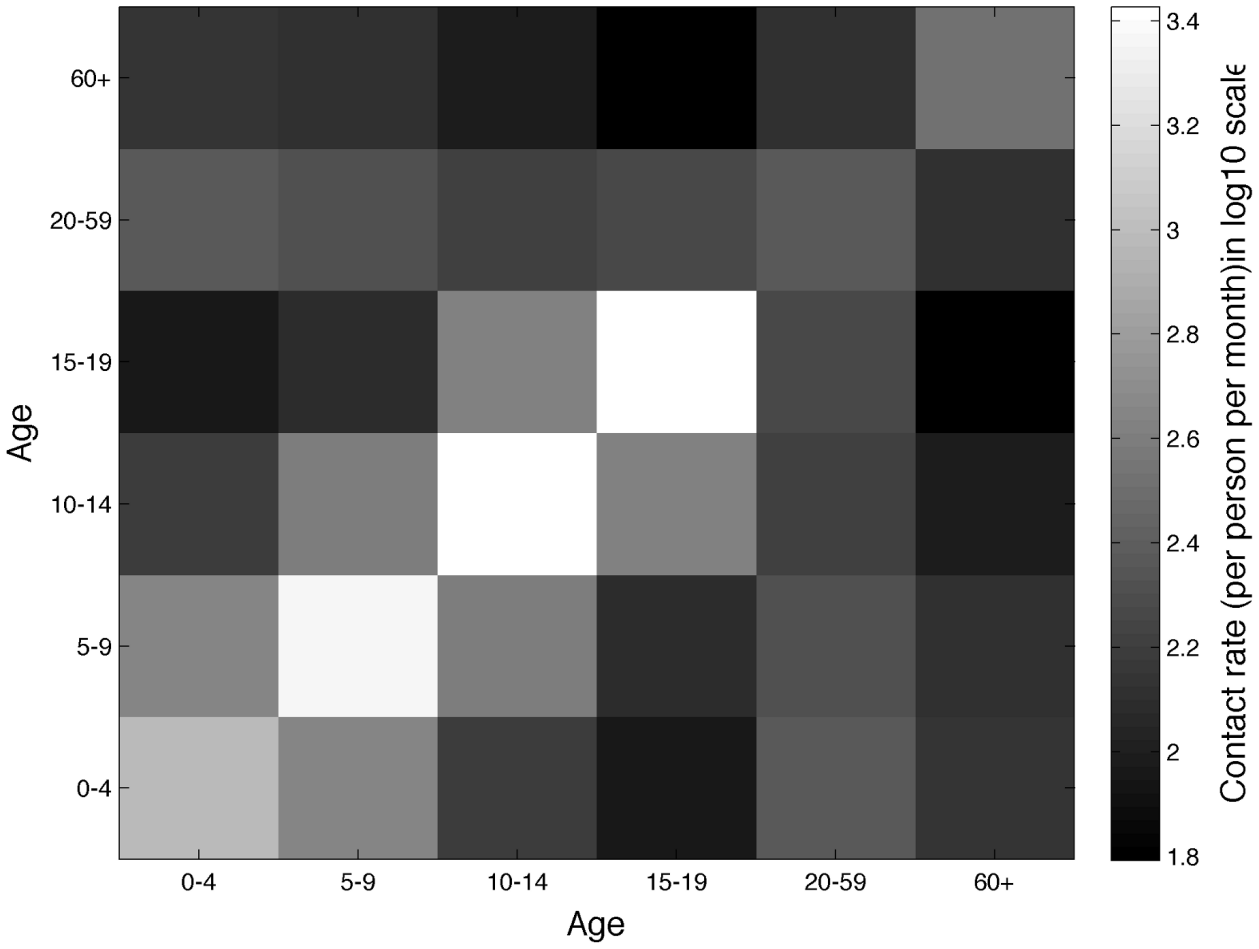
Monthly contact rates among age groups (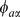). Calculated from survey data on physical contacts from Mossong et al. [25].

We stratified the population in our model into six age groups (years), 0–4, 5–9, 10–14, 15–19, 20–59, and 60+ yrs. The first four refined age groups are referred to as pediatric groups in this paper and are defined consistently with the age groups attending daycare/home-care, elementary school, junior high school, and high school in the US, respectively, as we are primarily interested in capturing the CA-MRSA transmission dynamics among young age groups. Contact rates across age groups were adapted from data on physical contacts previously derived from population surveys that were conducted in eight European countries [25] by collapsing the age groups 20–24, …, 55–59 into one adult group of age 20–59, and groups 60–64,65–69,70+ into a senior group 60+ yrs. following a similar approach as in [26] (Figure 2).

#### Epidemiological parameters

The basic epidemiological parameters and baseline values are listed in Table 1. Most of the initial (or most likely) values and ranges were obtained from a literature review while others were proposed by primary analysis of our epidemiological data. Specifically, we estimated the decolonization treatment coverage (or rate) (denoted as 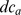, 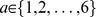) for each age group over the years by calculating the percentage of patients who were prescribed mupirocin (see Text S1 for details). The overall age-specific decolonization rate, 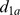, is then set to be the product of the treatment efficacy (percentage of successfully decolonized people among those who were prescribed mupirocin, denoted by 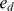 in Table 1), the age-specific treatment coverage 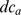 (values are given by Table S1), and the reciprocal of the duration of the drug effect (about 2 weeks [10]). Moreover, we assumed higher transition rates from colonized to infected states for both first-time and recurrent infections for individuals 0–4 and 15–19 yrs., as there was a significantly higher number of cases for these two groups in our epidemiological data, which is in agreement with other empirical studies[27], [28]. That is, 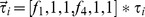 for 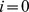 and 1, where 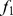 and 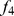 are the relative factors, and 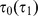 is the progression rate from colonized to infected for first-time (recurrent) infections for other age groups.

**Table 1:** Epidemiological parameters: definition, symbol, and initial value and range used in parameter estimation using the MCMC technique.

Definition, unit	Parameter	Initial Value	Range	Reference
probability of transmission per contact with infected	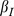	0.0017	0.0009–0.0025	[44], proposed
relative contagiousness factor for colonized to infected	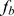	0.25	0–0.5	[24]
spontaneous decolonization rate, month^−1^	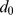	0.09	0.02–0.8	[45], [46]
efficacy of decolonization by mupirocin	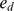	0.8	0.5–1	[47]
progression rate from colonized to infected (baseline), month^−1^	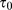	0.035	0.01–0.15	[24], [48]
relative risk of infection (from colonized) for 0–4 age group	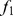	1.6	1.0–2.0	proposed
relative risk of infection (from colonized) for 15–19 age group	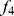	1.4	1.0–2.0	proposed
relative risk of progression rate from colonized to infected due to previous infection	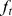	2.5	2.0–8.0	[49]
cure rate (1/(infection duration)), month^−1^	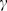	1	0.25–1.5	[18], [24]
immigration/migration rate, month^−1^	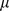	0.0029	-	[50]

‘Proposed’ in reference column means proposed by primary analysis of the data.

### Model calibration, validation and forecast

We calibrated our model given by System 0 with time series data of first time and recurrent infections for age groups 0–4, 5–9, 10–14, and 15–19 yrs. from January 2004 to December 2006 and estimated the unknown epidemiological parameters (Table 1). For this purpose, we assumed as initial conditions that all people in the population were free of past infections in January 2004 (first time point). That is, we set to 0 the initial value for 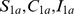, for 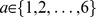. The initial number of people with first-time infections in adult groups, 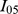 and 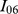, as well as the percentage of completely susceptible people in each age group at the beginning of 2004, 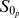 (common across the age groups), were taken as unknown parameters to be estimated.

We employed a delayed rejection adaptive Metropolis-Hastings (DRAM) algorithm in a Markov-Chain Monte-Carlo (MCMC) simulation framework [29] to estimate unknown model parameters. We used the widely-used MCMC package coded in Matlab (available from: http://helios.fmi.fi/~lainema/mcmc/) (Text S3). For each estimated parameter we assumed uniform prior distributions with range values as given in Table 1. Posterior distributions for each parameter were obtained from the resulting Markov chains [30]. Next, we selected a random sample of size 500 from the Markov chain of parameters to assess parameter uncertainty. For model validation, we compared our calibrated model forecast for two subsequent years of time series data covering the period 2007–2008.

Latin Hypercube Sampling (LHS) and Partial Rank Correlation Coefficient (PRCC) techniques [31] were used for sensitivity analysis to assess the impact of control strategies targeting different age groups on the reproduction number 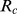. LHS provides remarkable efficiency in drawing a highly representative random sample of small size from a multi-dimensional distribution[32], [33]. The magnitude of PRCC quantifies the importance of individual parameters, with the sign of the PRCC value indicating the specific qualitative relationship between the input and the output variable. That is, positive values of PRCC implies that increasing values of the input variable lead to increasing values of the output variable. Since a PRCC indicates the degree of the monotonicity between a specific input variable and a specific output variable, only input variables that are monotonically related to the output variable are included in this analysis [32], [34]. Hence, we examined the scatterplots between 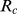 and each parameter to assess the monotonicity assumption.

We set out to analyze the effectiveness of the various types of control interventions including typical interventions targeting symptomatic infected individuals and novel decolonization strategies focused on the colonized reservoir pediatric population. Specifically, we evaluated reductions in contact rates by infected individuals due to personal awareness of infection. We also assessed the impact of decolonization treatment of infected people following regular antibiotic treatment of MRSA infections. Finally, we examined the possibility of disease elimination by hypothetical decolonization treatment strategies targeting colonized people (in compartment 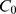). We modeled the first intervention by using an age-specific parameter, 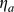, 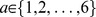, to denote the relative reduction in contact rates between different age groups. Thus, the force of infection given by Equation (1g) becomes
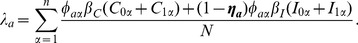
(2)The decolonization treatment targeting symptomatic infected individuals was modeled by varying the decolonization treatment coverage 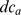 (in 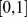), for 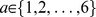. The effect of decolonization treatment for colonized people was modeled by adding an additional age-specific rate 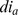, 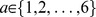, to the spontaneous decolonization rate 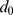, as the new transition rate from 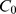 to 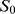 (Figure 1). Parameter 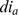 is the product of decolonization coverage (age-specific) and reciprocal of duration of drug effect (2 weeks [10], common across age groups) by assuming perfect drug efficacy. We estimated the associated reduction in 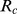 and determined whether infections approached zero as we varied the age-specific decolonization coverage.

## Results

### Model-based estimates

**Figure 3:**
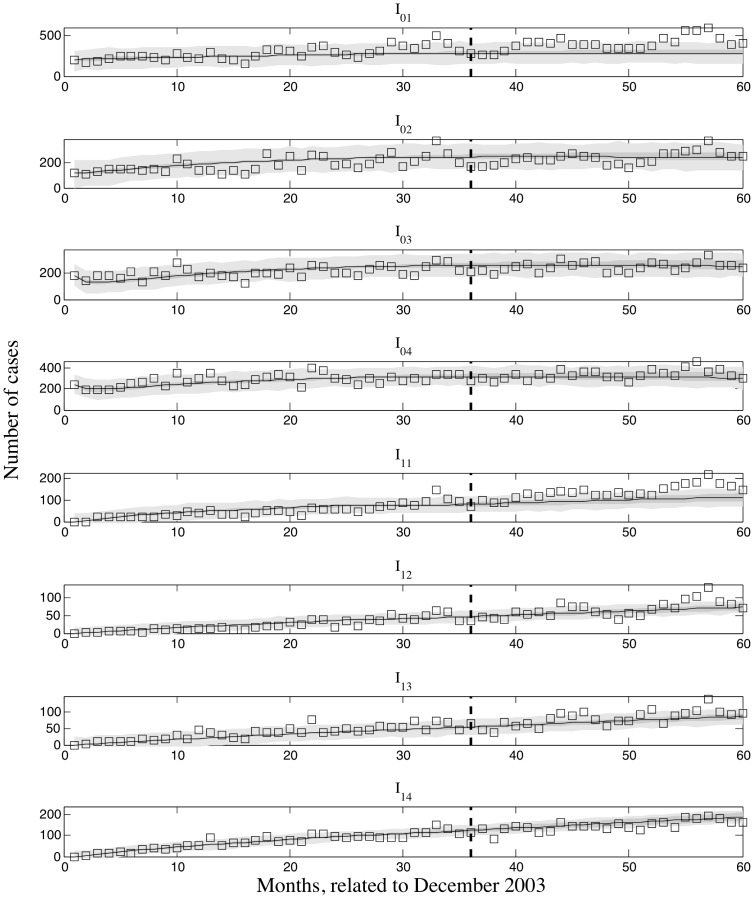
Model fit to MRSA infection data using MCMC toolbox. The wider band shows 95% probability limits for individual observations, the darker narrower band shows 95% probability limit for mean prediction and the black curve in the middle is the median prediction. Little squares are data points; the vertical dashed line indicates the calibration period (2004–2006) and the validation period (2007–2008).

Model parameter estimates and their corresponding geweke indices are shown in Table 2. Overall the model yielded a good fit to the incidence curve covering the period 2005–2006 albeit three of the parameter estimates did not achieve high convergence based on their geweke indices (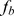, 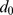 and 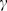) (Figure 3). Moreover, the model forecast for two subsequent years 2007–2008 tracked closely the additional incidence data.

**Table 2:** Statistics for estimated parameters based on the Markov chain generated by the adaptive Metropolis Hastings algorithm with 2004–2006 data.

Parameter	mean	std	geweke
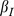	0.0021	0.00026	0.70
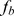	0.40	0.081	0.48
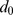	0.19	0.058	0.31
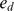	0.59	0.082	0.85
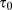	0.014	0.0017	0.78
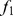	1.85	0.075	0.92
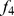	1.93	0.043	0.94
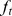	1.80	0.14	0.93
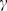	1.18	0.21	0.53
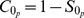	0.093	0.0047	0.92
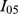	459	90	0.78
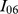	270	47	0.78

The geweke index closes to 1 means good convergence of the MCMC chain for that parameter.

### Uncertainty and sensitivity analysis for 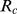


**Figure 4:**
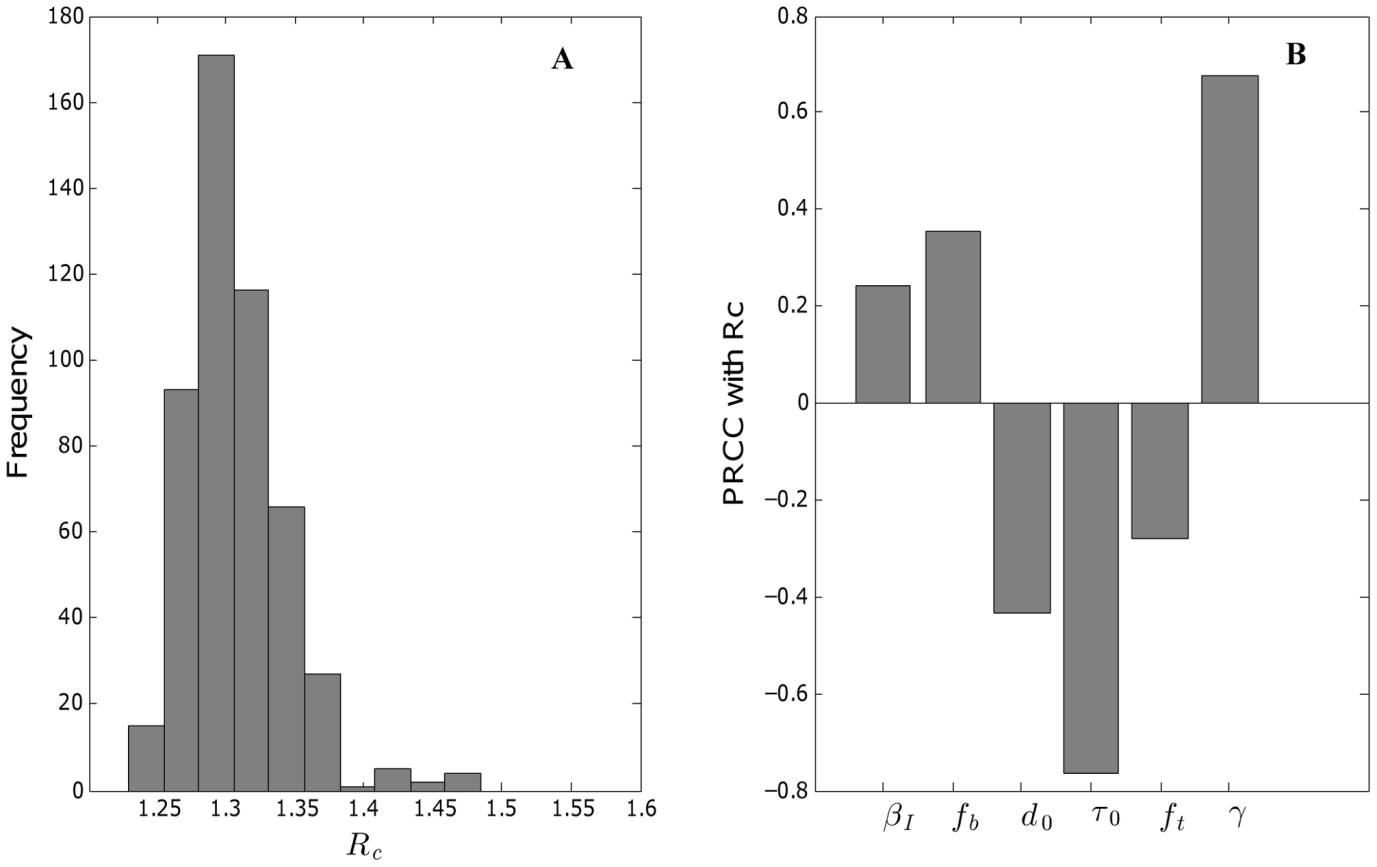
Uncertainty and sensitivity analyses on the control reproduction number 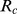. Histogram of 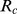 (A) and PRCC of parameters with 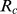 (B) with parameter uncertainty (based on a random sample of size 500 of Markov chain for parameters).

We estimated the control reproduction number 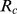 using Equation (S2). which we derived using the next generation matrix method [35], [36] (Text S2).Parameters 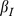, 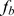, 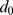, 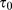, 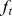 and 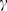 satisfied the monotonicity assumption and the corresponding PRCCs are shown in Figure 4B. We found that 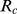 is most sensitive to the parameters 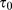 (progression rate from colonized to infected) followed by 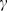 (reciprocal of the duration of being infected). 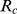 is also fairly sensitive to 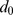 (spontaneous decolonization rate). All of them are significant from zero at the 0.001 level according to their p-values.

### Evaluating the effect of intervention strategies

#### The effectiveness of reducing contact rates by infected individuals

**Figure 5:**
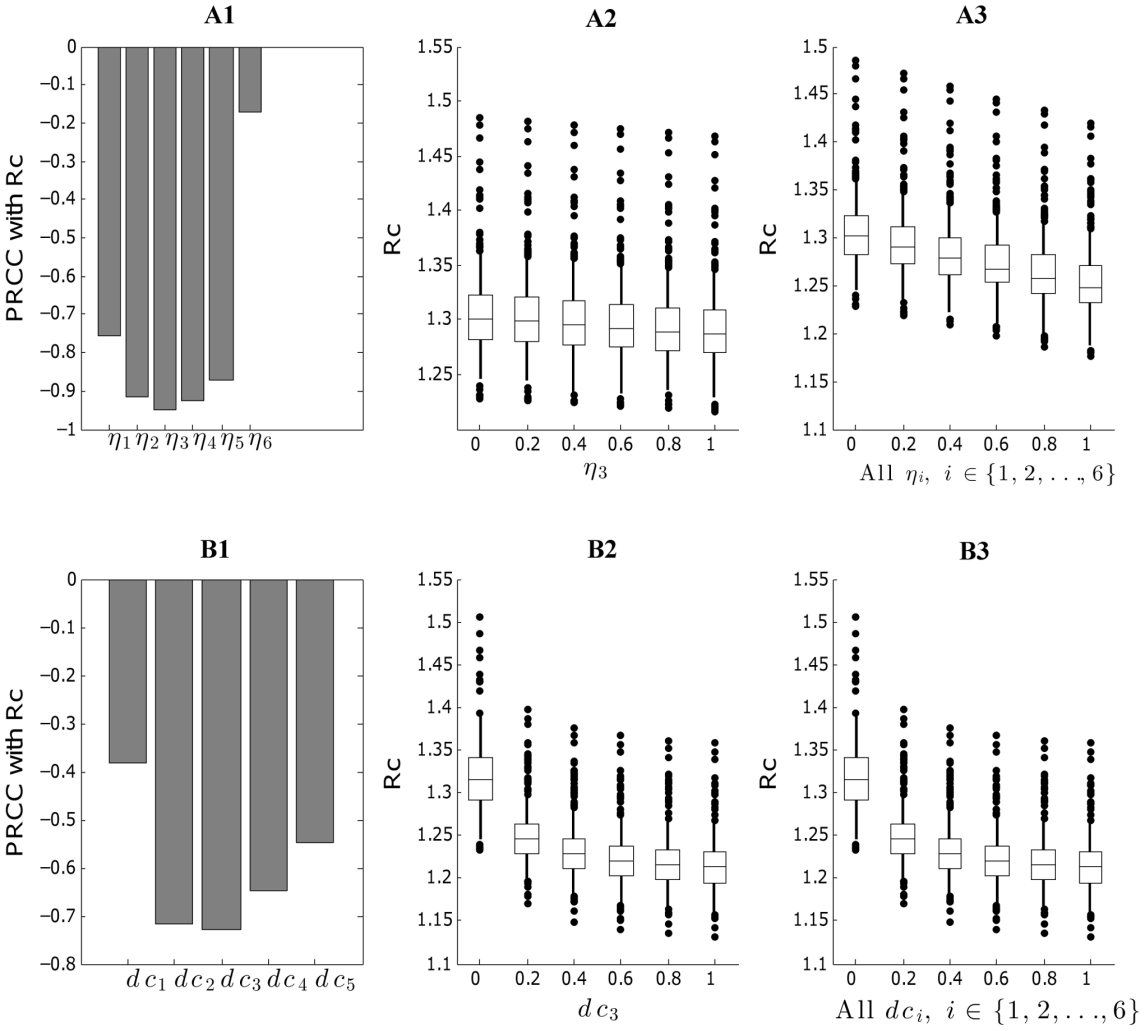
PRCC and 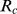 under different intervention strategies. Upper panel: PRCC of 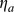 (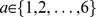, level of contact reduction in age group 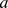), with 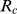 based on a sample of size 100 using LHS (A1); 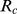 for different levels of contact reduction under uncertainty of other model parameters (A2); and 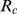 for different level of population-wide contact reduction under the uncertainty of other parameters(A3). Lower panel: PRCC of 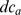 (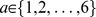, decolonization treatment coverage in age group 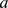), with 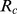 based on a sample of size 100 using LHS (B1); 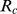 for different levels of decolonization treatment coverage under uncertainty of other model parameters (B2); and 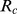 for different levels of population-wide decolonization treatment coverage under the uncertainty of other parameters (B3).

In order to assess the impact of contact rate reductions by infected individuals across different age groups, we used mean estimates of model parameters as given in Table 2. We drew a random sample of parameters 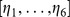 of size 100 from its 6-dimensional uniform distribution using LHS to calculate the distribution of 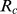 and the corresponding PRCCs of each of 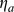
's. As expected, the greater the reduction in contact rate by infected individuals the higher the reduction in 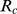, with the age group 10–14 yrs being slightly more influential on reducing 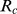 relative to other age groups. Specifically, we estimated the reduction levels in 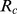 as we decreased the contact rates from 0 to 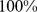 among infected individuals aged 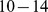 yrs (Figure 5A2) or across all age groups (Figure 5A3). For instance, mean 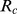 was reduced by only 1.0% when infected individuals aged 10–14 yrs did not make contacts with other individuals while 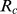 was reduced by 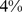 in the scenario that all infected individuals across all age groups did not make contacts with others.

#### Evaluating the effect of decolonization treatment on infected individuals

**Figure 6:**
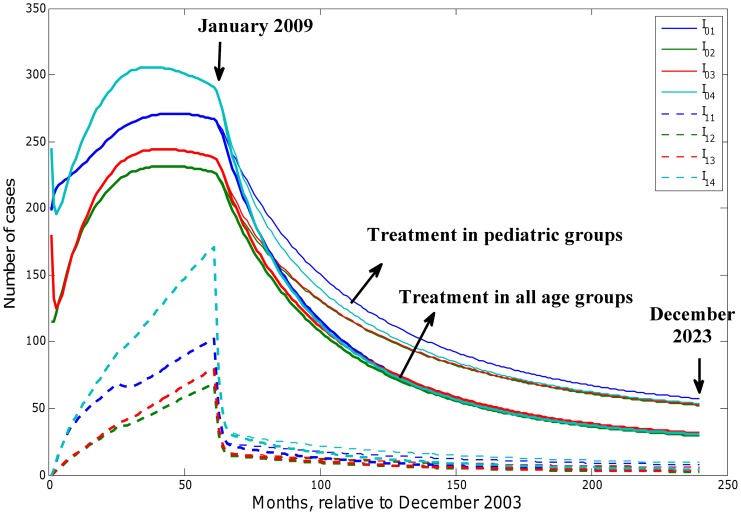
Model prediction for first-time and recurrent infections for pediatric groups until December 2023 with mean parameter values and different decolonization treatment coverage after 2008. 100% coverage for the first 4 age groups and 0 for adult groups (slim lines); 2). 100% coverage for the whole population (bold lines). The age-specific decolonization treatment coverage for till 2008 are time-dependent step functions with extreme values given by Table S1 for 4 pediatric groups and 0 for 2 adult groups.

To examine the effect of decolonization treatment strategies targeting symptomatic infected individuals, we quantified the sensitivity of each age-specific decolonization coverage 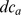 on 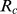. We found that All PRCCs across age groups for 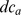
s followed the same pattern (Figure 5B1) except for 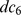, the decolonization treatment coverage for the 6th age group (60+ yrs.), which showed no monotonic relation with 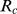 (not shown in Figure 5B1). The mean 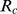 decreased by 2.1% when all infected individuals aged 10–14 yrs were treated by decolonization drugs for regular antibiotic treatment for CA-MRSA infections (Figure 5B2) while 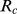 was reduced by 7.8% when all infected individuals across all age groups were treated by decolonization drugs (Figure 5B3). We found that decolonization treatment strategies targeting symptomatic infected individuals were not sufficient to achieve disease control. Simulations of decolonization treatment strategies targeting all infected individuals in pediatric age groups or across all age groups are shown in Figure 6. We found that recurrent infections were essentially brought under control, but first-time infections remained well above zero even when all infected individuals across all age groups underwent decolonization treatment.

#### Effectiveness of combinations of the two strategies targeting symptomatic infected people

**Figure 7:**
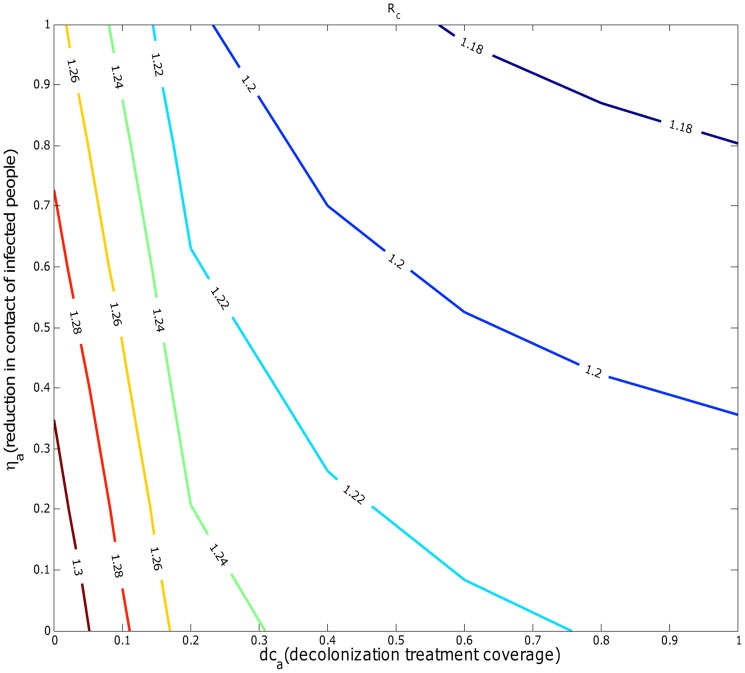
Mean value of 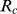 for a combined strategy of with different levels of reduction in contact of infected individuals (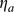) and decolonization treatment coverage the infected (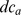) in all age groups under parameter uncertainty.

We assessed if a combination control strategy that includes reduction in contact rates by infected individuals with others and decolonization treatment for infected individuals was capable of achieving disease control. The effective values of 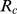 as a function of different reduction levels in contact rates by infected individuals and different levels of decolonization treatment coverage targeting symptomatic infected individuals across all age groups are shown in Figure 7. 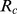 was no less than 1.18 under the combination of two strategies regardless of their intensity levels. In addition, the long term effect of single or combined intervention strategies on disease levels are illustrated in Table 3. It shows that decolonization treatment for the infected reduced much more total infections in the pediatric population than reducing contacts of infected people did, and a combination control strategy implementing both intervention strategies was not sufficient to achieve disease eradication.

**Table 3:** Model-based forecast of the number of first-time infections (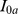)and recurrent infections (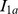) for the first age groups (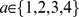), and reduction of total infections in the pediatric population (0–19 years) compared with baseline (no intervention) at the end of 2023 under different population-wide intervention strategies.

Strategy	*I*_01_	*I*_02_	*I*_03_	*I*_04_	*I*_11_	*I*_12_	*I*_13_	*I*_14_	total reduction
Baseline	150	133	137	143	182	146	172	312	0
Strategy 1	128	116	121	126	124	104	125	214	23%
Strategy 2	26	26	28	27	3	1	1	4	91%
Strategy 1 and 2	19	19	20	20	2	1	2	2	94%

Strategy 1: 100% reduction in contact of infected with others; Strategy 2: decolonization treatment for all infected.

#### Evaluating the impact of decolonization treatment strategies targeting colonized people

**Figure 8:**
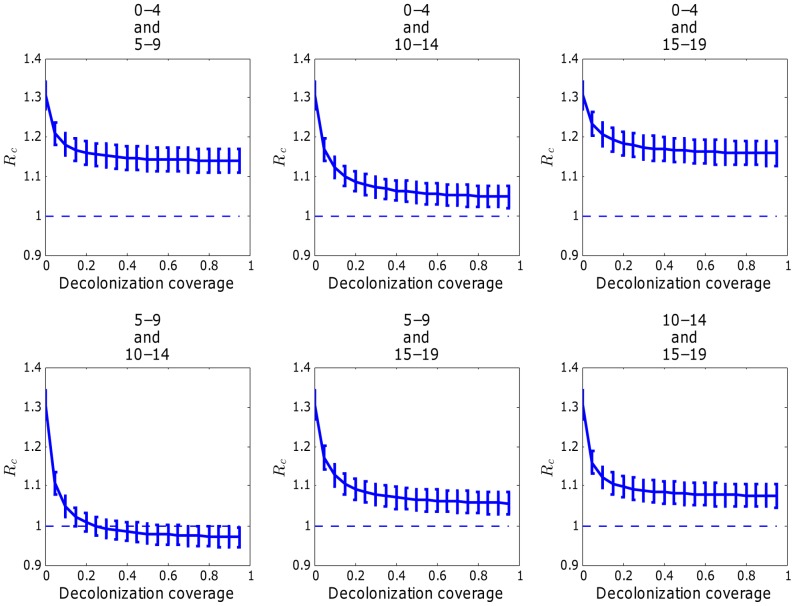
Mean 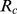 with different levels of decolonization treatment coverage for colonized people in various combinations of two of pediatric groups. The error bars are based on standard deviations of 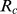
s under parameter uncertainty.

**Figure 9:**
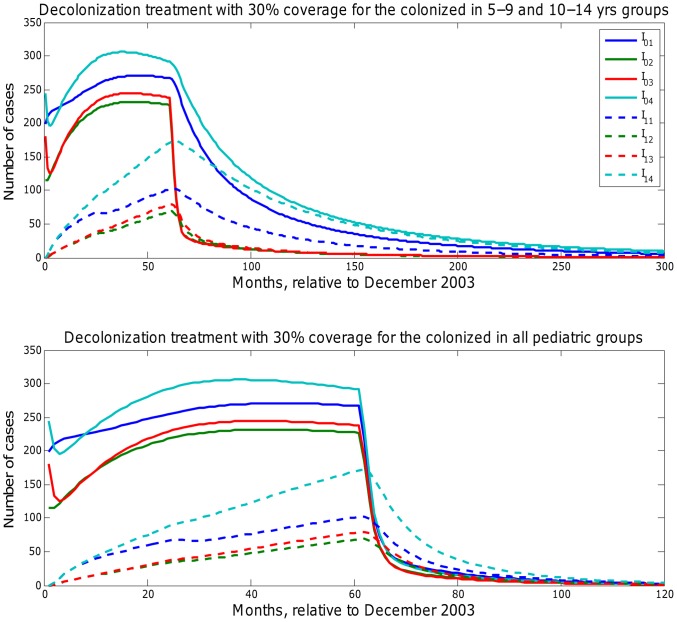
Long-term forecast of CA-MRSA infections in the pediatric groups under decolonization treatment strategy targeting colonized people with 30% coverage in different age groups. 5–9 and 10–14 yrs. age groups (upper panel); all pediatric groups (lower panel).

Whereas routine intervention strategies targeting symptomatic infected individuals were not found to be capable of achieving disease control, we also evaluated the impact of hypothetical intervention strategies targeting colonized (asymptomatic) individuals (compartment 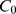 in Figure 1). We found that treatment of colonized asymptomatic individuals in any single age group was not enough to achieve disease control. However, by exploring decolonization treatment targeting combinations of two pediatric age groups, we found that decolonization treatment targeting colonized individuals in age groups 5–9 and 10–14 yrs. achieved 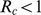 whenever coverage levels 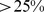 (Figure 8). By targeting colonized people across 4 pediatric age groups, we also found that the minimum decolonization treatment coverage necessary to achieve 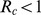 was 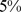. The long-term disease dynamics forecasts with decolonization treatment coverage at 30% implemented continuously starting in year 2009 on age groups 5–9 and 10–14 yrs. and across all pediatric groups are shown in Figure 9. Findings indicated that targeting individuals aged 5–14 yrs. required 20 years to achieve disease elimination while disease control could be achieved within 5 years when the intervention was focused on all pediatric groups.

## Discussion

We have developed and parameterized the first age-structured model of the transmission dynamics of MRSA transmission at the community level using data on skin and soft tissue infections in children and teenagers who were enrolled in the Medicaid Program in Maricopa County, Arizona. Our compartmental epidemic model includes age heterogeneity in contact rates, infectiousness, and decolonization treatment rates, and keeps track of individuals with past infection history. We estimated the control reproduction number at 1.3 with 95% confidence interval [1.2, 1.4]. Sensitivity analysis of 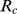 on the model parameters revealed that 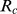 is most sensitive to the parameters 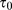 (progression rate from colonized to infected) followed by 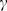 (reciprocal of the duration of being infected) and 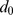 (spontaneous decolonization rate). Using our calibrated model, we found that typical strategies focused on infected individuals were not capable of achieving disease control when implemented alone or in combination. In contrast, our results suggest that novel decolonization strategies that target the general pediatric population colonized with CA-MRSA have the potential of achieving disease elimination.

We performed numerical simulations to explore the impact of various feasible intervention strategies such as reductions in contact rates by infected people owing to personal awareness of infection and effect of decolonization treatment targeting symptomatic infected individuals belonging to specific age groups or across the entire population. We found that neither a single or combined strategy was able to achieve 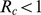. We also forecasted short-term disease dynamics in the presence of both types of interventions strategies starting in 2009. Our results suggest that reductions in contact rates by infected people has little effect on the disease prevalence, and that neither of the two strategies was capable of achieving disease control particularly among first-time infections. Given that these two intervention strategies are the most widely practiced (intuitively and clinically), our model-based results provide an explanation to the persistent levels of CA-MRSA in many US regions over 20 years since its first appearance [3]–[6]. We also tested the effectiveness of some hypothetical decolonization treatment strategies targeting asymptomatic colonized people. We found that 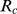 could be reduced below 1 when the treatment was focused on 5–9 and 10–14 yrs. age groups with coverage at 25%, and an even smaller coverage (5%) was sufficient when all pediatric groups groups were targeted. Long-term forecast of the infections showed that disease elimination is feasible within 5 years through decolonization treatment at 30% of colonized people in the pediatric population starting in 2009.

Compared with published models on MRSA transmission, our model is novel in several ways. First, our model accounts for transmission in a heterogeneous population based on age-specific contact rates calibrated using survey data on physical contacts [25]. This enables our model to capture much more essential elements of the complex MRSA transmission problem and test the effect of different control strategies that target different age groups. Second, our model was calibrated and provided a good fit to time series data using solid estimation techniques rather than solely relying on assumed parameter values from the literature. It is worth mentioning that our estimate of the control reproduction number at 1.3 is in line with that assumed in prior studies (e.g., [37]). Moreover, it important to note that while our model incorporates key features of the transmission process including a realistic population contact structure and dominant features of MRSA epidemiology, there are many limitations to policy models with respect to the incorporation of bio-medical, operational, political, and economic features. No one model can claim to incorporate all assumptions and features given the limited data available to calibrate them. We believe our transmission model could be useful to evaluate further control scenarios and formulate rational policy based on the best available evidence.

There are limitations to note about our model calibration. Some of the Markov chains did not converge perfectly within 10000 steps in terms of the geweke index. However, our objective here was to find reasonable values of parameters so that our model captured qualitatively the overall trend of the age-specific time series data. In this sense, the MCMC algorithm worked much better than other sampling methods (e.g., Latin Hypercube Sampling or simple random sampling with repetition). Moreover, we also calculated the R-squared(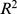), a widely-used index for goodness of fit for a general model, for each of the eight output variables, and it turned out that the 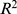
s for first-time infections were much poorer (less than 0.5) than those for the recurrent infections (greater than 0.7). Hence, our model did a better job in modeling recurrent infections than first time infections. Of note, our model described by System (1) does not account for seasonality patterns [38], which may explain a significant fraction of variation in data that remained unexplained by the model. Moreover, we did not account for the changing medical practice as our understanding of this pathogen has increased since 2004. The infection duration decreased over these years as the appropriate antibiotics changed from second line medications to first line medications [39]–[41]. Therefore the model prediction might have overestimated the infection prevalence.

Our dataset itself has several limitations. First, our data correspond to general SSTIs which may not be directly related to CA-MRSA. However, it has been observed from microbiological analyses that the fraction of MRSA-related SSTIs remained stationary among the total SSTIs in our study population for our study period [24]. Given that it will be difficult to truly determine the exact cases of MRSA-related SSTIs and their microbiological spectrum at the population level, our data set is the best among those available. Second, the target population of the data set corresponds to the children and young adults in the Medicaid program in Maricopa County, AZ. It is important to note that patients under Medicaid coverage belong to a lower income population, where the rate of infection has been described to be higher than that of the general population[5], [42]. Hence, our model prediction likely overestimates the prevalence in the overall population in Maricopa County. Further studies are needed to shed light on whether our findings can be generalized to other settings.

In summary, our model indicates that intervention strategies targeting only infected people, either by reducing their contact frequency with healthy people or by pharmaceutical decolonization are not capable of eliminating CA-MRSA infections at the population level. By contrast, substantial reductions in the prevalence of HA-MRSA could be achieved via contact reductions via patient isolation, enhanced hand hygiene and screening and health-care worker cohorting strategies (e.g., [43]). Our control scenarios based on decolonization treatment strategies that target asymptomatic colonized individuals indicate that finding a cost-effective method to locate colonized individuals and conducting decolonization treatment on a limited fraction of them could prove to be an effective intervention. However, the possibility of resistance emerging from increased use of mupirocin cannot be overemphasized. Future modeling studies could be carried out to to evaluate the effect of strategies aimed at reducing the impact of resistance emerging from large-scale use of mupirocin. Moreover, further work should focus on the collection of morbidity data and quantification of the attack rates and transmission dynamics of CA-MRSA in other world settings with different socio-economic and climatic characteristics. While our transmission model is based on key epidemiological features of CA-MRSA including age-specific heterogeneity in contact rates, infectiousness and decolonization treatment rates, predictions from more elaborate models that incorporate, for instance, seasonality in transmission efficiency or a more detailed picture of the transmission process (e.g., household level transmission models) could be undertaken in future work as relevant epidemiological data become available.

## Supporting Information

Dataset S1 Data set used for model calibration and validation.
(XLS)

Table S1 Average decolonization treatment rate estimated for two time periods from variable ‘rx’ in Dataset S1.
(PDF)

Text S1 Description of Dataset S1 and data manipulation.
(PDF)

Text S2Mathematical deduction of control reproduction number (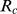).(PDF)

Text S3 Description of model calibration using MCMC technique.
(PDF)
