# Supplementary material for: A Data-Driven Mathematical Model of CA-MRSA Transmission among Age Groups: Evaluating the Effect of Control Interventions
Source: PLoS Comput Biol. 2013 Nov 21;9(11):e1003328. doi: 10.1371/journal.pcbi.1003328 (PMC3836697; doi:10.1371/journal.pcbi.1003328)
Supplement: Table S1 — Average decolonization treatment rate estimated for two time periods from variable ‘rx’ in Dataset S1. (PDF) [file pcbi.1003328.s002.pdf]

Table S1. Average decolonization treatment rate estimated for two time periods

	Decolonization treatment rate: mean (std)	
Age group (years)	2004-2005	2006-2008
0-4	0.0315(0.0169)	0.0851(0.0244)
5-9	0.0237(0.0143)	0.0585(0.0199)
10-14	0.0199(0.0131)	0.0454(0.0182)
15-19	0.0173(0.0106)	0.0396(0.0173)
