# Supplementary material for: A Data-Driven Mathematical Model of CA-MRSA Transmission among Age Groups: Evaluating the Effect of Control Interventions
Source: PLoS Comput Biol. 2013 Nov 21;9(11):e1003328. doi: 10.1371/journal.pcbi.1003328 (PMC3836697; doi:10.1371/journal.pcbi.1003328)
Supplement: Text S1 — Description of Dataset S1 and data manipulation. (PDF) [file pcbi.1003328.s003.pdf]

# Data Description

Our data set comprises 58803 observations and 16 variables including 'case id', 'instance', 'begin month', 'begin year', 'age' and 'rx'. Specifically, the variable 'instance' (=1, 2, 3, 4, 5 or 6) records the number of infections for a patient, that is, 1= first time infection, and 2 = second time infection. We take those with 'instance' $\geq 2$ as recurrence infections. By distinguishing infection type (first time or recurrent by 'instance') and age groups (0-4, 5-9, 10-14, 15-19 years, by 'age'), we calculated time series of monthly cases for first time infection (corresponding to $I_0$ in the model represented by Figure 1) and recurrent infection (corresponding to $I_1$) for each of the four age groups. These are monthly absolute number of SSTI cases. Since it was observed that MRSA-related infection is a major cause and make a stationary percentage of SSTIs [1], we used SSTI time series data for model calibration.

The variable 'rx' indicates whether mupirocin was prescribed during the visit. We used this to parameterize decolonization treatment rate. We observed that the age-specific decolonization treatment rate was stable in 2004-2005, then jumped up suddenly to another value for 2006-2008. This was true for all age groups. We calculated the average for each of the time periods for each age group (Table S1).

We also obtained the total number of people in each age group enrolled in Medicaid program in Maricopa County in each year of 2004-2008. The population distribution in this data set is triangle-shaped with more young children. We rescaled the infection data according to population distribution in Maricopa County in 2004 based on US Census data [2]. For each data point in a monthly time series of infection for an age group, the rescaled number of cases were obtained according to the following equation:

$$\frac{\text{No. of Cases from data}}{\text{Rescaled No. of cases}} = \frac{\text{Population size from data}}{\text{Population size from US Census}}$$

The rescaled data of infections ($I_0$, $I_1$ for the first 4 age groups) for years 2004-2006 was used for model calibration and years 2007-2008 for model validation. We kept the population of the first age group (0-4 years), but rescaled the other groups' according to the ratio shown by US Census data in 2004. Populations remained approximately unchanged over the years of interest [2]. Hence we took them constants to facilitate computational convenience.

# References


1. Panchanathan SS, Petitti DB, Fridsma DB (2010) The development and validation of a simulation tool for health policy decision making. Journal of Biomedical Informatics 43: 602–607.

2. Bureau USC (2004). Population data. URL `http://www.census.gov/popest`.
