# Supplementary material for: A Data-Driven Mathematical Model of CA-MRSA Transmission among Age Groups: Evaluating the Effect of Control Interventions
Source: PLoS Comput Biol. 2013 Nov 21;9(11):e1003328. doi: 10.1371/journal.pcbi.1003328 (PMC3836697; doi:10.1371/journal.pcbi.1003328)
Supplement: Text S2 — Mathematical deduction of control reproduction number (). (PDF) [file pcbi.1003328.s004.pdf]

Control reproduction number and reproductive value

Instead of using *basic reproduction number*, R_0, to show how many people on average can be infected by an infected individual over the course of infectious period in a totally susceptible population [1] , we use *control reproduction number*, R_c, to represent the same quantity for a system with control (treatment of infected).

As in [2], System (1)with λ_a given by Equation (1g) can be written more compactly in vector notation as

$$\frac{d\vec{S_0}}{dt} = -\vec{\lambda} \otimes \vec{S_0} + \vec{d_0} \otimes \vec{C_0} + \vec{\mu} \otimes \vec{N} - \vec{\mu} \otimes \vec{S_0}, \tag{S1a}$$

$$\frac{d\vec{C_0}}{dt} = \vec{\lambda} \otimes \vec{S_0} - \vec{d_0} \otimes \vec{C_0} - \vec{\tau_0} \otimes \vec{C_0} - \vec{\mu} \otimes \vec{C_0}, \tag{S1b}$$

$$\frac{d\vec{I_0}}{dt} = \vec{\tau_0} \otimes \vec{C_0} - \vec{\gamma} \otimes \vec{I_0} - \vec{\mu} \otimes \vec{I_0}, \tag{S1c}$$

$$\frac{d\vec{S_1}}{dt} = -\vec{\lambda} \otimes \vec{S_1} + (\vec{d_0} + \vec{d_1}) \otimes \vec{C_1} - \vec{\mu} \otimes \vec{S_1}, \tag{S1d}$$

$$\frac{d\vec{C_1}}{dt} = \vec{\gamma} \otimes (\vec{I_0} + \vec{I_1}) + \vec{\lambda} \otimes \vec{S_1} - (\vec{d_0} + \vec{d_1}) \otimes \vec{C_1} - \vec{\tau_1} \otimes \vec{C_1} - \vec{\mu} \otimes \vec{C_1}, \tag{S1e}$$

$$\frac{d\vec{I_1}}{dt} = \vec{\tau_1} \otimes \vec{C_1} - \vec{\gamma} \otimes \vec{I_1} - \vec{\mu} \otimes \vec{I_1} \tag{S1f}$$

with

$$\vec{\lambda} = \frac{1}{N}\phi(\beta_C(\vec{C_0} + \vec{C_1}) + \beta_I(1 - \vec{\eta}) \otimes (\vec{I_0} + \vec{I_1})), \tag{S1g}$$

The vectors are column vectors, for example, $\vec{S_0} = (S_{01}, \ldots, S_{06})^T$, and $\otimes$ in the equations represents component-wise product for matrices/vectors.

Using the next generation matrix technique [3, 4], we have

$$\mathbf{x} = \begin{bmatrix} \vec{C_0} \\ \vec{I_0} \\ \vec{C_1} \\ \vec{I_1} \end{bmatrix}, \quad \mathcal{F} = \begin{bmatrix} \vec{\lambda} \otimes \vec{S_0} \\ \mathbf{0} \\ \vec{\lambda} \otimes \vec{S_1} \\ \mathbf{0} \end{bmatrix}, \quad \mathcal{W} = \begin{bmatrix} (\vec{d_0} + \vec{\tau_0} + \vec{\mu}) \otimes \vec{C_0} \\ -\vec{\tau_0} \otimes \vec{C_0} + (\vec{\gamma} + \vec{\mu}) \otimes \vec{I_0} \\ -\vec{\gamma} \otimes (\vec{I_0} + \vec{I_1}) + (\vec{d_0} + \vec{d_1} + \vec{\tau_1} + \vec{\mu}) \otimes \vec{C_1} \\ -\vec{\tau_1} \otimes \vec{C_1} + (\vec{\gamma} + \vec{\mu}) \otimes \vec{I_1} \end{bmatrix}.$$

We assume that all susceptible have no past infections at the beginning of the disease, i.e., $\vec{S_0} > 0$, $\vec{S_1} = 0$. Then,

$$\mathbf{F} = \begin{bmatrix} \mathbf{F_C} & \mathbf{F_I} & \mathbf{F_C} & \mathbf{F_I} \\ \mathbf{0} & \mathbf{0} & \mathbf{0} & \mathbf{0} \\ \mathbf{0} & \mathbf{0} & \mathbf{0} & \mathbf{0} \\ \mathbf{0} & \mathbf{0} & \mathbf{0} & \mathbf{0} \end{bmatrix},$$

with

$$\mathbf{F_C} = \frac{\beta_C}{N}\phi \otimes (\vec{S_0}\vec{1}^T),$$

and

$$\mathbf{F_I} = \frac{\beta_I}{N}\phi \otimes (((1 - \vec{\eta}) \otimes \vec{S_0})\vec{1}^T),$$

where $\vec{1} = (1,1,1,1,1,1)^T$; and

$$\mathbf{W} = \begin{bmatrix} \mathbf{W}_{\vec{d_0}+\vec{\tau_0}+\vec{\mu}} & \mathbf{0} & \mathbf{0} & \mathbf{0} \\ -\mathbf{W}_{\vec{\tau_0}} & \mathbf{W}_{\vec{\gamma}+\vec{\mu}} & \mathbf{0} & \mathbf{0} \\ \mathbf{0} & -\mathbf{W}_{\vec{\gamma}} & \mathbf{W}_{\vec{d_0}+\vec{d_1}+\vec{\tau_1}+\vec{\mu}} & \mathbf{0} \\ \mathbf{0} & \mathbf{0} & -\mathbf{W}_{\vec{\tau_1}} & \mathbf{W}_{\vec{\gamma}+\vec{\mu}} \end{bmatrix},$$

with

$$\mathbf{W}_{\vec{d_0}} = diag(\vec{d_0}), \text{ etc..}$$

By matrix calculation, we have

$$\mathbf{FW^{-1}} = \begin{bmatrix} \mathbf{A} & \mathbf{B} \\ \mathbf{0_1} & \mathbf{0_2} \end{bmatrix},$$

where

$$\begin{aligned} \mathbf{A} = \mathbf{W^{-1}}&_{(\vec{d_0}+\vec{\tau_0}+\vec{\mu})(\vec{\gamma}+\vec{\mu})[(\vec{\gamma}+\vec{\mu})(\vec{d_0}+\vec{d_1}+\vec{\tau_1}+\vec{\mu})-\vec{\gamma}\vec{\tau_1}]} \\ &[\mathbf{W}_{(\vec{\gamma}+\vec{\mu})[(\vec{\gamma}+\vec{\mu})(\vec{d_0}+\vec{d_1}+\vec{\tau_1}+\vec{\mu})-\vec{\gamma}(\vec{\tau_1}-\vec{\tau_0})]}\mathbf{F_C} \\ &\mathbf{W}_{\vec{\tau_0}(\vec{\gamma}+\vec{\mu})(\vec{d_0}+\vec{d_1}+\vec{\tau_1}+\vec{\mu})}\mathbf{F_I}], \end{aligned}$$

$\mathbf{B}$ is a nonzero matrix, $\mathbf{0_1}$ and $\mathbf{0_2}$ are zero matrices with proper dimensions such that $\mathbf{FW^{-1}}$ has the same dimension as $\mathbf{F}$. This gives

$$R_c = \rho(\mathbf{FW^{-1}}) = \rho(\mathbf{A}). \tag{S2}$$

To compare the relative contributions of infectious people in each age group to an outbreak, we used the concept of reproductive value from population biology. It is given by the left eigenvector corresponding to the eigenvalue R_c of the next generation matrix $\mathbf{FW^{-1}}$ [5]:

$$\vec{v}^T(\mathbf{FW^{-1}}) = R_c\vec{v}^T,$$

or

$$(\mathbf{FW^{-1}})^{\mathbf{T}}\vec{v} = R_c\vec{v}.$$

The reproductive value is only a measure of the contributions of the age groups at the beginning of an outbreak.

References

1. Macdonald G (1961) Epidemiologic models in studies of vetor-borne diseases: The re dyer lecture. Public health reports 76: 753.

2. Medlock J, Galvani AP (2009) Optimizing influenza vaccine distribution. Science 325: 1705–1708.

3. Diekmann O, Dietz K, Heesterbeek J (1991) The basic reproduction ratio for sexually transmitted diseases: I. theoretical considerations. Mathematical biosciences 107: 325–339.

4. Van den Driessche P, Watmough J (2002) Reproduction numbers and sub-threshold endemic equilibria for compartmental models of disease transmission. Mathematical biosciences 180: 29–48.

5. Caswell H (2006) Matrix population models. Wiley Online Library.
