# Supplementary material for: A Data-Driven Mathematical Model of CA-MRSA Transmission among Age Groups: Evaluating the Effect of Control Interventions
Source: PLoS Comput Biol. 2013 Nov 21;9(11):e1003328. doi: 10.1371/journal.pcbi.1003328 (PMC3836697; doi:10.1371/journal.pcbi.1003328)
Supplement: Text S3 — Description of model calibration using MCMC technique. (PDF) [file pcbi.1003328.s005.pdf]

Model calibration by MCMC algorithm

The function given by the file *mcmcrun.m* in the toolbox employs delayed rejection and/or adaptive Metropolic-Hastings (M-H) algorithm [1–4] and produces Markov chains to represent the underlying joint posterior distribution of parameters for nonlinear Gaussian models. In our simulation, the variances of the eight measured components, $I_{0a}, I_{1a},\ a \in \{1,2,3,4\}$, were given by inverse gamma distribution with parameters $(S^2_{0_{ia}}, N_{0_{ia}}), i \in \{1,2\}, a \in \{1,2,\ldots,4\}$, respectively, with S_0^2s being the initial error variances and to be updated by the inverse gamma distribution. We set $S^2_{0_{ia}} = 10$ for $i = 1$ and 5 for $i = 2$ for all values of a, and $N_{0_{ia}} = 4$. The prior distribution for each unknown parameter was set to be uniform with proper range (Table 1). For the other three, we set $S_{0_p} \in (.9, .99)$ (or $C_{0_p} = 1 - S_{0_p} \in (.01, .1)$), $S_{05} \in (400, 600)$ and $S_{06} \in (200, 400)$.

We allowed the algorithm to run for 10000 iterations with burn-in of 5000 iterations, and the geweke convergence diagnostic method was employed to assess convergence of chains [5]. Geweke value closes to 1 indicates good convergence of a chain.

References

1. Haario H, Saksman E, Tamminen J (1999) Adaptive proposal distribution for random walk metropolis algorithm. Computational statistics 14: 375–395.

2. Haario H, Saksman E, Tamminen J (2001) An adaptive metropolis algorithm. Bernoulli 7: 223–242.

3. Tierney L, Mira A (1999) Some adaptive monte carlo methods for bayesian inference. Statistics in Medicine 18: 2507–2515.

4. Mira A (2001) Ordering and improving the performance of monte carlo markov chains. Statistical science 16: 340–350.

5. Geweke J (1992) Evaluating the accuracy of sampling-based approaches to calculating posterior moments. In: JM Bernardo AD J Berger, Smith A, editors, Bayesian Statistics 4, Oxford: Oxford University Press.
